# COGNITIVE PERFORMANCE OF STUNTED PRE-SCHOOL CHILDREN UNDERGOING NUTRITIONAL RECOVERY TREATMENT

**DOI:** 10.1590/1984-0462/;2018;36;1;00007

**Published:** 2017-11-13

**Authors:** Thaíse Morais Silva, Nassib Bezerra Bueno, Maria de Lourdes da Silva Gomes de Azevedo, Ana Paula Grotti Clemente, Telma Maria de Menezes Toledo Florêncio

**Affiliations:** a Faculdade de Nutrição da Universidade Federal de Alagoas, Maceió, AL, Brasil.; b Universidade Tiradentes, Maceió, AL, Brasil.

**Keywords:** Malnutrition, Nutrition dwarfism, Child development, Desnutrição, Nanismo nutricional, Desenvolvimento infantil

## Abstract

**Objective:**

To determine if the treatment of stunted children offered at a specialized center influences their cognitive performance.

**Methods:**

Two groups of children from vulnerable families were selected, one consisting of stunted children being treated at the Nutrition Education and Recovery Center (CREN), and the other group of eutrophic children from a local, public day care center. At CREN, children are treated in a day-hospital system (9 hours/day, 5 days/week), receiving medical, nutritional and psycho-pedagogical support. All children were submitted to the Denver-II Development Screening Test and had their development and the height-for-age index assessed at 3 moments: at the beginning of the follow-up, and after 6 and 12 months. The socioeconomic status, according to the Brazilian Economic Classification Criteria, was assessed at the beginning of the follow-up. Data were treated by prevalence ratios for cross-sectional baseline analysis, using the Poisson regression, and by pooled prevalence ratios for longitudinal analysis, using a generalized equation estimation model, both adjusted by age, sex and economic status.

**Results:**

Seventy-four children were included, 37 for each group. There were no differences in age, sex and socioeconomic status between groups. In the longitudinal analysis, the CREN group showed better performance in the personal-social domain (pooled prevalence ratio: 0.89; 95% confidence interval - 95%IC 0.82-0.95), with no differences in the other domains.

**Conclusions:**

The treatment offered at CREN satisfactorily improved the social skills of the treated children, without changing other domains.

## RESUMO

**Objetivo:**

Determinar se o tratamento de crianças com baixa estatura, ofertado em um centro especializado, influencia seu desempenho cognitivo.

**Métodos:**

Foram analisados dois grupos de crianças advindas de famílias vulneráveis, um com crianças com baixa estatura em tratamento no Centro de Recuperação e Educação Nutricional (CREN) e outro de crianças eutróficas de uma creche municipal localizada na mesma região do CREN. No CREN, as crianças são tratadas em semi-internato (9 horas/dia, 5 dias/semana), recebendo suporte médico, nutricional e psicopedagógico. Todas foram submetidas ao Teste de Triagem do Desenvolvimento de Denver-II e avaliadas quanto ao índice de estatura-para-idade em 3 momentos distintos: no início do acompanhamento e após 6 e 12 meses. A classificação socioeconômica das crianças, de acordo com os Critérios de Classificação Econômica Brasil, foi feita no início do acompanhamento. Calcularam-se razões de prevalência para a análise transversal da linha de base, por meio de uma regressão de Poisson, e razões de prevalência agrupadas para a análise longitudinal, por meio de um modelo de estimativas de equações generalizadas, ambas ajustadas por idade, sexo e classe socioeconômica.

**Resultados:**

Ao todo, 74 crianças foram analisadas, 37 em cada grupo. Não houve diferenças de idade, sexo e classe socioeconômica entre os grupos. Na análise longitudinal, o grupo CREN apresentou melhor desempenho no domínio pessoal-social (razão de prevalência agrupada: 0,89; intervalo de confiança de 95% - IC95%: 0,82-0,95), sem diferenças significativas para os demais domínios.

**Conclusão:**

O tratamento ofertado pelo CREN melhorou satisfatoriamente as habilidades sociais das crianças tratadas, sem alterar os demais domínios.

## INTRODUCTION

The full development of a child depends on his or her genetic potential and on environmental, cultural, and social factors to which he or she is exposed.^1^ The most critical period are the first one thousand days of life (from conception to the second birthday), when growth and the development of the nervous system are intense.^2^ However, in the second childhood and at school age, the physical and motor aptitudes present more evolution, since in this stage children become aware of themselves and the world around them, accomplishing fast independence, social adaptation and development, with learning advances.^3^

In the learning process, cognitive development is characterized by a few domains of interdependent functions, such as gross motor skills, which refer to the use of major body muscles; fine motor skills, which are related with the use of minor hand muscles; language, which is important for problem resolution and for taking attitudes; and personal-social development, which refers to the processes of accomplishing independence to conduct daily activities.^4^ The identification of possible risks of developmental and growth delay for the child should be diagnosed as early as possible; therefore, the impact will be lower, and the intervention, more effective.^5^

For that, there are many instruments used to assess, quantify and monitor intellectual development.^6^ The Denver-II developmental screening test stands out among the main methods (DDSTII), which is easy to apply and can be used by any professional in the health field. It is a screening test that evaluates all four areas of development: social-personal, fine motor, language and gross motor.^7^

Growth, as an important instrument to determine the infant health status, is influenced by the environment in which the children live and interferes directly in their development.^8^ Socioeconomic level and family context work as mediators for the proper intake of nutrients, which, added to the occurrence of diseases, affect the children’s nutritional status.^9^ The malnourished children present with cognitive development delay, which may lead to individual and collective long term consequences.^10^ However, the recovery of the growth deficit in children may soften the effects of malnutrition on cognitive performance, so that the recovered children present with a level of cognition similar to that of those who do not have such deficit.^11^

Aiming at fighting growth deficit and its negative impacts on communities with high social vulnerability, the Nutrition Education and Recovery Centers (CREN) were created. These are non-profit organizations related with the Federal Universities of São Paulo (UNIFESP) and Alagoas (UFAL). In CREN, the children with height deficit are cared for in a semi boarding school format, staying in the location nine hours a day, five days a week, receiving medical, nutritional, psychological and pedagogical care.^12^

Therefore, this study aimed at assessing if the treatment provided by the Nutrition Education and Recovery Center in Maceió-AL (CREN-AL), specialized in the recovery of height deficit among preschoolers, coming from vulnerable socioeconomic classes, influences their cognitive performance in comparison to children with adequate height coming from the same social reality, enrolled in a daycare facility from the municipal network for one year.

## METHOD

The Research Ethics Committee from Centro de Estudos Superiores de Maceió - CESMAS (COEPE) approved the project with protocol n. 1588/12. The legal tutors authorized the participation of the children by signing a Consent Form, besides the Assent form for the minors and the consent from the people in charge of the institutions in which data was collected.

This is a longitudinal study that lasted for 1 year and had 3 moments of collection (beginning, after 6 months and after 12 months), which accompanied preschoolers (2 to 5 years) enrolled in two institutions in the city of Maceió, Alagoas: CREN-AL, and in a municipal daycare facility close to CREN. These institutions are located in the 7^th^ administrative region of Maceió - the one with the lowest human development index in the city.

In CREN, children with height deficit remained in the semi-boarding school regime, from 8 a.m. to 5 p.m., and had 5 meals, which provided them with 80% of the daily energetic needs, aiming at recovering such deficit. CREN offers medical and nutritional care for the children, besides developing pedagogical activities that are adequate to the age group, monitored by a multiprofessional team including a psychologist and an educationist. In the municipal daycare facility, the children remain for one shift, from 8 a.m. to 12 p.m., and have the school lunch, besides pedagogical follow-up according to the age group.

The selection of children was carried out with a nutritional status diagnosis. It included children from CREN with low stature, that is, with Z-score for height-to-age (H/A) <-2 standard deviations (SD), and at least three months of hospitalization. In the daycare only eutrophic children were included, that is, with Z-score for H/A <-2. Children who presented with any neurological disorders previously diagnosed were excluded.

The children were assessed as to their height, in the 3 moments of the study, by using a stadiometer with an inextensible metric tape, 2 m long and with a 0.1 cm precision to calculate the H/A index, using the software AnthroPlus, version 2007 (World Health Organization, Geneve, Switzerland). The categorization of the economic class was conducted only in the beginning of follow-up, using the Brazilian Economic Classification Criterion (CCEB), from the Brazilian Association of Research Institutions (ABEP).^13^

The evaluation of the cognitive development of children was conducted with DDSTII.7 This test assesses four areas/categories: gross motor (body motor control, how to sit and walk), fine motor adaptive (eye-hand coordination, manipulation of small objects), language (production of sound, ability to recognize, understand and use the language) and personal-social (aspects regarding the socialization of the child inside and outside the family environment). It is comprised of 105 items that demonstrate tasks related with the field it approaches and presents the classification as follows: normal, delay and non-testable.

According to the evaluation criteria, children classified as “normal” were the ones who had at most one failure in execution per investigated area. The children who had two or more failures were classified with “delay”, and those who refused to execute any items, preventing the appreciation of their performance in one or more fields, were classified as “non-testable”.

The data were analyzed in the software Statistical Package for the Social Sciences (SPSS), version 20.0 (IBM SPSS Inc, Chicago, IL, USA). The continuous data are presented as mean and SD, and categorical data as absolute and relative frequencies. In the beginning of follow-up, to compare the continuous variables (age and H/A) between groups, the prerequisite of homogeneity of variance was evaluated by the Levene test, and, if found, the Student’s t test was conducted for independent samples; as for the comparison of categorical variables (sex and economic class) between groups, the chi-squared test was used.

To verify the association between cognitive development (normal or delayed) in the four areas of DDSTII and the groups (CREN or daycare facility), in the cross-sectional analysis, in each one of the three moments, prevalence ratios (PR) were calculated using the Poisson regression model, with robust estimation of variance, adjusted for age, socioeconomic class and sex. In the longitudinal analysis, a model of generalized estimating equation, with an independent correlation matrix (GENLIN command) - also adjusted for age, socioeconomic class and sex - was built to incorporate the intra-subject variability of repeated measures, generating a clustered estimation of PR between groups (CREN and daycare facility) and cognitive development (normal or delayed) for each one of the four DDSTII domains. Alpha equal to 5% was established for all analyses.

## RESULTS

Seventy-four children participated in the study: 37 attending CREN and 37 attending the municipal daycare facility. The socioeconomic and anthropometric characteristics can be seen in Table 1. There were no differences regarding age, sex and socioeconomic class between the groups. As expected, the CREN group presented significantly lower values for the H/A index. The average performance in the 4 DDSTII domains, in the beginning of follow-up, was of 44% in children in the municipal daycare facility, and 41% in the CREN group. During the follow-up period, the CREN group recovered, in average, 0.4 Z-score of the H/A index, whereas in the daycare group the increase was of 0.1 Z-score.

**Table 1:** Socioeconomic and anthropometric characteristics of the two groups of children analyzed.

	CREN (n=37)		Daycare facility (n=37)		p-value^**a**^
	Mean	Standard deviation	Mean	Standard deviation	
Age (months)	42.0	11.0	44.9	9.4	0.35
H/A index (Z-score)	-2.4	0.3	-0.3	0.5	<0.01
	n	%	n	%	p-value^b^
Social Class					
C1-C2	14	37.8	18	48.6	0.34
D-E	23	62.2	19	51.4	
Sex					
Female	18	48.6	18	48.6	0.99
Male	19	51.4	19	51.4	

CREN: Nutrition Education and Recovery Center; H/A: height-to-age; ^a^obtained using the Student’s t test for independente samples; ^b^obtained using the chi-squared test.

**Figure 1:**
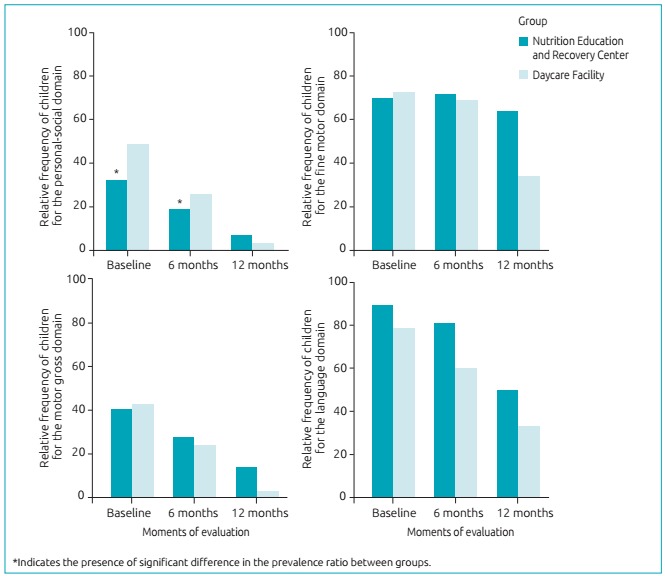
Relative frequency of the “delay” score for the groups Nutrition Education/Recovery Center and Daycare Facility, in the three moments of evaluation.

Table 2 shows the longitudinal analysis of the performance in DDSTII for the daycare facility and CREN groups, and the clustered PR for each domain, obtained through a model of generalized estimating equations, adjusted by age, social class and sex. According to this analysis, only the personal-social domain presented differences between the groups with time: the risk of the CREN group presenting a “delay” score was lower than the risk in the daycare facility: clustered PR=0.89; 96% confidence interval (96%CI) 0.82-0.95; p<0.01. Figure 1 shows the non-adjusted prevalence of “delay” scores for each domain in DDSTII throughout the three moments for the CREN and daycare facility groups. It is possible to verify that only the personal-social domain showed significant differences between the groups during the follow-up period.

**Table 2:** Evaluation of the cognitive development of children from the Nutrition Education and Recovery Center and a municipal daycare facility along 12 months.

Evaluation	Initial			6 months			12 months			Clustered analysis		
	CREN	Mun.		CREN	Mun.		CREN	Mun.				
Domain	%	%	PR^**a**^	%	%	PR^**a**^	%	%	PR^**a**^	PR^**b**^	95%CI	p-value
Personal-social	32.4	48.6	0.85^c^	18.8	25.7	0.86^c^	7.1	3.0	1.01	0.89	0.82-0.95	<0.01
Fine motor	70.3	73.0	1.01	71.9	68.6	1.00	64.3	36.4	1.08	1.03	0.95-1.12	0.43
Gross motor	40.5	42.9	0.99	28.1	24.3	0.97	14.3	3.0	1.07	1.01	0.91-1.12	0.78
Language	89.2	78.4	1.07	81.3	60.0	1.06	50.0	33.3	0.98	1.05	0.98-1.13	0.15
Untestable	27.0	43.2	0.85^c^	34.4	22.9	0.99	21.4	12.1	1.00	0.94	0.85-1.03	0.21

CREN: Nutrition Education and Recovery Center; Mun: Municipal daycare facility; PR: prevalence ratio; 95%CI: 95% confidence interval; ^a^prevalence ratio in the CREN group by presenting the result “delay” in relation to the daycare facility group, calculated with the Poisson regression with robust variance estimation, adjusted by sex, age and social class; ^b^clustered prevalence ration in the CREN group by presenting the result “delay” in relation to the daycare facility group throughout the three moments, obtained by a generalized equation estimation, adjusted for age, sex, and social class; ^c^these prevalence ratios presented p-value<0.05 with the Poisson regression.

## DISCUSSION

Generally, the general cognitive performance in the four domains of the DDSTII in children from vulnerable socioeconomic classes, with low stature or normal stature, was low, since the group of children with low stature undergoing nutritional treatment in CREN presented about 41% of improvement. This number is a bit different from the group of eutrophic children in the same region (44%), in the beginning of follow-up. By doing a longitudinal comparison of this cognitive performance throughout a year, it was possible to observe that the personal-social domain was different between groups, and that children from the CREN group presented with lower risk of having a “delay” score. For the other categories, there were no differences between groups.

The findings in this study suggest that the relationship between cognitive performance and nutritional status of children is influenced by the social environment in which they live. Studies with vulnerable preschoolers show that lower socioeconomic status is damaging for the cognitive development of the children.^14^ ^,^ ^15^ ^,^ ^16^ ^,^ ^17^

Children with low stature did not present significant differences in any domain when compared to eutrophic children in the same community, as observed by Saccani et al.,15 who evaluated two groups of children (malnourished and eutrophic) living in the suburbs of Porto Alegre, Rio Grande do Sul, and showed that the nutritional status was not related with the learning performance of the children. Even though it is a known fact that the adequate infant nutrition is essential for the performance of children’s potentials and abilities, it is possible to infer that besides nutrition, the sum of social and environmental factors can overcome the biological factors, thus causing deficit in intellectual capacity, regardless of the nutritional status. One investigation with children with low stature in the first grade of elementary school, in the suburbs of São Paulo, showed that they presented worse performance in school in comparison to children without low stature, emphasizing that nutritional recovery should happen early in order to prevent negative effects in the future learning process.^18^

Children with low stature undergoing nutritional treatment in CREN presented better performance in the personal-social domain, in comparison to those in the daycare facility. This result may be influenced by the fact that children in the semi-boarding regime in CREN spent two shifts in the institution, with adequate pedagogical support, which is more intense than that provided by the daycare facility, where children spend only one shift. Assuming both groups live in an unhealthy domestic environment, with low stimulation at home - due to the low schooling level of the parents -, it is possible to justify the absence of differences in the evolution of cognitive performance in the other areas between the groups.

Both groups presented high percentage of delay in some domains of DDSTII, especially regarding the language domain, as observed by Biscegli et al.,^19^ who assessed children enrolled in daycare facilities from Catanduva, São Paulo, with low socioeconomic status, and showed that language was the area with worse performance.

It is possible that the sample size did not ensure sufficient statistical power to find significant differences between the groups. However, the use of a longitudinal data analysis, using generalized estimating equation models, allows to minimize the sample loss throughout the study by considering all data available about the individuals.

The authors conclude that, among the children from vulnerable socioeconomic classes, there are no differences regarding the cognitive performance of those with low stature submitted to nutritional recovery treatment and the eutrophic ones. Children submitted to treatment in CREN presented better performance in the personal-social domain, possibly due to the semi-boarding model to which they are submitted.
